# First Aid to the Injured

**Published:** 1884-06

**Authors:** 


### First Aid to the Injured.

One of our funny exchanges gives the following:

1. Bites of all sorts are painful, and if not treated with expedition and skill, they sometimes prove very dangerous. The most common kinds are those received from dogs, mosquitoes, and bears. The rarest kinds are trilobites and jacobites.

2. One seldom, if ever, gets a bite when out fishing.

3. If about to be bitten by a dog, while serenading or foraging in a melon patch, immediately take some violent exercise, in order to preserve a good circulation. For instance, run a mile or so without stopping.

4. Never stop running because there is a man with a club apparently chasing the dog—sometimes he is encouraging him.

5. If this does not accelerate the action of the heart, climb the nearest tree.

6. Don't get down again for the purpose of rescuing the sample of your trousers. This is one of the dog's perquisites, and he wants it for his scrap-book.

